# A New Approach to EMG Analysis of Closed-Circuit Movements Such as the Flat Bench Press

**DOI:** 10.3390/sports6020027

**Published:** 2018-03-28

**Authors:** Artur Golas, Adam Maszczyk, Petr Stastny, Michal Wilk, Krzysztof Ficek, Robert George Lockie, Adam Zajac

**Affiliations:** 1 Department of Theory and Practice of Sport, The Jerzy Kukuczka Academy of Physical Education, 40065 Katowice, Poland; a.golas@awf.katowice.pl (A.G.); a.maszczyk@awf.katowice.pl (A.M.); m.wilk@awf.katowice.pl (M.W.); ficekk@poland.com (K.F.); a.zajac@awf.katowice.pl (A.Z.); 2 Department of Sport Games, Faculty of Physical Education and Sport, Charles University, 16252 Prague, Czech Republic; 3 Department of Kinesiology, California State University, Fullerton, CA 92831, USA; rlockie@fullerton.edu

**Keywords:** electromyography, elite sport, case study, powerlifting, resistance training

## Abstract

Background: The bench press (BP) is a complex exercise demanding high neuromuscular activity. Therefore, the main objective of this study was to identify the patterns of muscular activity of the prime movers on both sides of an elite powerlifter. Methods: A World Champion (RAW PR 320 kg) participated in the study (age: 34 years; body mass: 103 kg; body height 1.72 m; one-repetition maximum (1 RM) flat bench press: 220 kg). The subject performed one repetition of the flat bench press with: 70% 1 RM (150 kg) and 90% 1 RM (200 kg) in tempos: 2 s eccentric and 1 s concentric phase; 6 s eccentric and 1 s concentric phase). The activity was recorded for: pectoralis major, anterior deltoid, and triceps brachii (lateral and long head). Results: The total sum of peak muscle activity for the four analyzed muscles during both phases of the BP with the different loads and tempos was significantly different, and greater on the right side of the body. Conclusions: The use of lighter loads activate muscle groups in a different activation level, allowing for a greater muscle control. Lifting submaximal and maximal loads causes an activation of most motor units involved in the movement. Experienced athletes have a stabilized neuromuscular pattern for lifting which has different bilateral activity contribution.

## 1. Introduction

The bench press (BP) is a complex upper body exercise in which substantial external loads can be lifted, demanding high neuromuscular activity. The potential of the BP for strength development and popularity of BP competitions have made it a unique phenomenon as a popular exercise for training, testing or research purposes [1]. Previous studies have examined the: kinematics of the bench press movement [2]; effect of different chest press exercises [3]; unstable surfaces [4]; impact of fatigue [5], as well as successful and unsuccessful attempts [6]; and different approaches in the bench press. These have included comparisons of concentric-only bench press to one performed with a countermovement [7], or comparison of isometric versus dynamic bench press exercises [2], and the performance analysis of the BP and chest press exercises with maximal and submaximal loads [8].

Many studies concerning the topography of muscle strength describe how particular groups of muscles contribute to total strength [9]. The neuromuscular recruitment has been assessed only by EMG amplitude to observe the influence of exercise load as an external stimulus for greater muscular development [10,11]. There are essentially five major categories of data that can be derived from EMG analysis [12]: muscle activity; degree of muscle activity; when the muscle switches ‘on’ or ‘off’; how much is the muscle active; and degree of fatigue. The tonic aspect of neurophysiologic behavior of motor units during muscular contraction, which is related to intensity of muscular activation, needs to be initially considered [1,9,10]. The central nervous system (CNS) is responsible for processing information received from the environment (via the sensory cortex) and commanding a response from the rest of the body. Neural pathways that are well used and developed are retained and promoted, whereas those that are less needed in the present situation will be pruned or shut down to enable the release of brain capacity [13]. The neuromuscular system uses two strategies to regulate the amount of force generated while performing a certain task. The first strategy includes motor unit recruitment (i.e., calling into play more motor units and muscle fibers), while the other is rate coding (i.e., how rapidly electrical impulses, or action potentials, are fired down the motor neuron to the muscle fiber it innervates) [13]. 

The traditional muscle model for the BP was based on prediction by biomechanical lever arms and empirical knowledge, where the pectoralis major (PM), anterior deltoid (AD) and triceps brachii (TB) were estimated as the prime movers. This specific muscle model was first applied by Elliott [14]. More recent studies have revealed that the neuromuscular activity during the BP is dependent not only on the co-activation of prime movers, but also on the activation of their antagonist muscles as stabilizers [1]. Despite numerous research conducted with the flat BP, most authors have based their conclusions on EMG analysis from one side of the body. In the traditional approach, EMG measurements and analysis are conducted on the prime movers, on the dominant side of the body in accordance with the SENIAM procedure [1,15,16,17]. According to the authors of this paper this can be a serious limitation in the interpretation of results, especially in elite athletes. 

In strength athletes at the elite level, excessive training loads cause numerous injures which require changes in neuromuscular patterns during resistance exercises. In symmetrical exercises such as the BP or the barbell squat, these changes may occur between the left and right limb, making evaluation from one side of the body incomplete. This could lead to erroneous conclusions if the EMG analysis is only based on one side of the body. 

Therefore, this study investigated a new approach to the EMG analysis of closed-circuit movement such as the BP. The main objective of this study was to identify the patterns of muscular activity of the prime movers on both sides of the body. Muscle involvement in the lift will be expressed by the contribution of each muscle separately as well as the total activity of the four studied muscles (PM, AD, TBlat, TBlong), both in the eccentric and concentric phases of the flat bench press. Considering our preliminary research with EMG analysis of the bench press in experienced powerlifters, we have hypothesized that there will be significant differences in neuromuscular activity of the prime movers on the left and right side of the body. 

## 2. Materials and Methods

### 2.1. Participant

One athlete, World Champion in RAW (bench press with no additional equipment, just protective tolls such as lifting belt) professional BP with best lift 320 kg participated in the study (age: 34 years; body mass: 103 kg; body height 1.72 m; one-repetition maximum (1 RM) classic flat bench press: 220 kg). The participant did not perform any additional resistance exercises for 72 h prior to testing to avoid fatigue. The subject was informed verbally and in writing about the procedures and possible risks and benefits of the tests and provided written consent before the research commenced. The study received the approval of the Bioethics Committee at the Academy of Physical Education in Katowice, Poland (NRSA 404054). 

### 2.2. Procedures

The measurements were performed in the Laboratory of Muscular Strength and Power at the Academy of Physical Education in Katowice. There were two sessions of the experiment. Session 1 involved the determination of the 1 RM flat bench press. Session 2 consisted of the subject performing one repetition of the flat bench press with two loads: 70% 1 RM (150 kg) and 90% 1 RM (200 kg) with different time under tension (TUT; 2 s eccentric and 1 s concentric phase—2/1; 6 s eccentric and 1 s concentric phase—6/1). Rest periods between sets equaled 5 min. A standardized warm-up protocol was used for each session, including a general warm-up (5 min), using a hand cycle ergometer (heart rate of approximately 130 bpm) and several lower and upper body resistance exercises (split squats, squats, push-ups, bench press pulls). The specific part of the warm-up consisted of three bench press sets with the load adjusted accordingly to perform 15 (at 60% of 1 RM), 10 (at 70% of 1 RM) and 5 (at 80% of 1 RM) repetitions.

The determination of the 1 RM was performed according to the protocol by [5]. The percentage of the 1 RM load was calculated based on the self-reported values by the participant. The self-reported 1 RM was set according to the information given by the participant on maximal lifts performed in the previous three months. The rest periods between sets were 5 min to avoid the potential effects of fatigue. When the self-reported 1 RM was successful, a trial with an additional load of 2.5–5 kg was performed. When the initial trial was unsuccessful, the weight was decreased by 2.5–5 kg. A total of 2 trials were performed by the study participant. Two experienced spotters assisted the athlete in the preload phases.

### 2.3. Electromyography

An eight-channel Noraxon TeleMyo 2400 system (Noraxon USA Inc., Scottsdale, AZ, USA; 1500 Hz) was used for recording and analysis of biopotentials from the muscles. The activity was recorded for four muscles: pectoralis major (PM), anterior deltoid (AD), and triceps brachii (lateral—Tblat; and long—Tblong head). Before placing the gel coated self-adhesive electrodes (Dri-Stick Silver circular sEMG Electrodes AE-131, NeuroDyne Medical, Cambridge, MA, USA), the skin was shaved, abraded, and washed with alcohol. The electrodes (11 mm contact diameter and a 2 cm center-to-center distance) were placed along the presumed direction of the underlying muscle fiber according to the recommendations by SENIAM [7,12]. The EMG signals were sampled at a rate of 1000 Hz. Signals were band pass filtered with a cut off frequency of 8 Hz and 450 Hz, after which the root-mean-square (RMS) was calculated. Electrodes were located on muscles on the left and right side of the participant. The grounding electrode was placed on the connection with the anterior deltoid. A video record was used for identification of the beginning and completion of the movement. After completion of all the tests in a single day, 2–3 s tests of isometric exercise were performed in order to normalize electromyographic records according to SENIAM procedure [7]. The normalization procedure was conducted for each side of the body separately (to estimate peak maximum voluntary contraction values—MVIC, %). The TB MVIC was obtained during lying triceps extension with 90° elbow flexion, the AD MVIC at 90° seated arm flexion, and the PM MVIC during an isometric bench press at 90° elbow flexion. All MVIC tests were performed against a fixed multi-press bar [17,18]. Analysis was based on peak activity during the bench press (both from the eccentric and concentric phases). Furthermore, the mean submaximal dynamic voluntary contraction from 70% 1 RM (MVIC_dyn-submax_) at 2 s/1 s lift was calculated [19] for other observed conditions. The MVIC_dyn-submax_ is normalization method, where the mean submaximal dynamic voluntary contraction is used as the basic (100%) for normalizing EMG values at different conditions. 

### 2.4. Data Analysis

The peak muscle values were analyzed descriptively, where 20% of MVIC in fixed basis indexes difference was considered a significant change in muscle activity [20]. Moreover, the 20% of MVIC change often approximates the standard deviation value in interindividual measurement during strength exercises [20,21]. Besides individual muscles analyses, total muscle activity (as the sum of all four muscle peaks) was evaluated. The evaluation was made between eccentric and concentric phases of the movement, between different loads (70% and 90% 1 RM), between the right and left side of the body and between two times of tension (2 s/1 s and 6 s/1 s) using indexes with fixed basis. Dynamics variability (MVIC_dyn-submax_) in fixed basis indexes with as a sum of four muscles during the eccentric and concentric phase of the flat bench press with different times of tension (2/1 and 6/1) and right and left side of the body were analyzed, where 20% difference was considered as a significant muscle activity difference [20,22]. 

## 3. Results

Changes in peak muscle activity for the four analyzed muscles during the eccentric and concentric phases of the flat BP with the different loads and TUT are presented in Table 1. The fixed index values differed between both the eccentric and concentric phases of the flat BP movement. The sum of peak muscle activity for the four analyzed muscles during both phases of the BP with the different loads and TUT were different (with exception of 70% 1 RM during 2 s/1 s TUT), and greater on the right side of the body. The right and left side of the body difference in peak activity between the loads, difference between exercise TUT, and differences between both types of contraction occurred in each measured muscle (at least in one inter-relation). The heavier load on the right side on the body exceed the 100% of MVIC during the concentric phase in all muscles (with the exception of TB long head). On the left side of the body only AD values exceeded 100% of MVIC during the concentric phase at 90% 1 RM.

As stated, the sum of mean muscle activities of all muscles was used to compare the MVIC_dyn-submax_, which was expressed as a percentage and is shown in Table 2. The MVIC_dyn-submax_ in total muscle activity differed between right and left side of the body during both phases of the lift (eccentric and concentric) at the resistance of 90% 1 RM. 

## 4. Discussion

The main finding of this study is that the peak muscle activity for the four analyzed muscles during the eccentric and concentric flat bench press with loads of 70% and 90% 1 RM during two different times under tension (TUT; 2/1 and 6/1) is significantly different between the left and right side of the body. The sum of MVIC activity on the dominant (right) side was higher in comparison to the non-dominant (left) side, which is determined by different involvement of particular muscle groups in the bench press movement. This phenomenon is most relevant in the concentric phase of the movement at the highest load (200 kg). On the non-dominant side of the body, the ADpeak reached the greatest MVIC. On the dominant side of the body the activity of ADpeak significantly differentiated the involvement of the remaining three prime movers. 

Considering these results, it is important to understand that the production and increased application of forces is the result of neuromuscular processes in response to a command from the CNS. Movement is a coordinated response to the actions of the sensory and motor nervous systems. An EMG measurement shows the action potential generated in the recruited motor units of the muscle examined [23]. As the resistance increases in a particular movement, a concomitant rise in the amplitude of the electromyographic signal occurs [24]. Theoretically, the greater external load the higher the activation of the muscle involved in the movement. Our case study shows that this is true only for the total activity of all the studied muscles, but not for particular muscles involved in the bench press movement. On the non-dominant side of the body an increase in external load from 150 to 200 kg caused a stagnation or even a decrease in triceps brachii activity (TBlatpeak, TBlongpeak), which was compensated by significantly greater activity of the ADpeak.

The changes in activity of particular prime movers due to variation in external load and TUT may be partially attributed to the sport technique of the individual athlete which has been developed and optimized throughout many years of training. A well-developed sports technique places the joints in the correct position and moves them in the best possible sequence to optimize the anatomical arrangement of the muscles. A mastered technique indicates that the chosen muscles (i.e., those muscles that have evolved to best undertake this task) will be recruited and in position to perform the work required [25]. An interventional study that observed the movement patterns of resistance trained athletes found minor differences in muscle activity on repeated EMG measurements [1,26], suggesting that elite athletes exhibit repeatable muscle activation patterns in different tasks. The same was true for our case study participant, who was an elite, very experienced power-lifter. The participant showed little differences in neuromuscular activity of the prime movers during the bench press movement in three consecutive seasons (unpublished data). Besides the above mentioned factors, training experience and sports level, phase of the annual training cycle, injures, applied training means and methods significantly determine neuromuscular control [27].

In the eccentric phase of the flat bench press movement, peak muscle activity at 70% 1 RM (TUT 2 s/1 s and 6 s/1 s) for both sides of the body was greater for TBlatpeak. At 90% 1 RM (TUT 2 s/1 s and 6 s/1 s) the ADpeak showed the greatest activation on both sides of the body. During the concentric phase of the movement at both loads, the greatest activity was registered for the ADpeak, the smallest prime mover. Our results confirmed that the optimal use of BP technique can be utilized only at maximal and submaximal loads, when the objective is to successfully complete the exercise [28]. According to Sakamoto and Sinclair [29], once a strategy set by the central nervous system to perform a motor task is chosen, it is implemented by activation of a group of muscles in the appropriate sequence. The selection of the correct muscles to be activated is simplified by certain principles [1,24]. One of these principles is directed at optimizing muscle coordination to minimize energy expenditure. Another principle is related to the prediction of forces, such as gravity or inertial interactions among body segments [30]. Considering our results, we assume that the patterns of muscle activity on the left and right side are more similar when lifting higher loads. When using lighter loads during the concentric phase of the bench press movement, the CNS can activate individual muscle groups in a different order between particular sides of the body. 

In our study, we have confirmed the activity of the prime movers during the flat bench which is consistent with numerous references [1]. However, our study is one of the first to indicate that the internal movement structure of the flat BP is significantly different between the right and left side of the body depending on the load and time under tension (2 s/1 s; 6 s/1 s). Another interesting finding is the fact that the longer eccentric phase of the movement at 90% 1 RM reduced muscle tension in the concentric phase, which indicated the creation of better conditions for the lift. This can be attributed to more elastic energy cumulated in the prolong eccentric phase of the movement, in succession use in the concentric phase of the lift. 

The MVIC_dyn-submax_ in the eccentric phase of the flat bench press movement on the right side of the body showed the greatest increase of activity at 90% 1 RM load. The left upper limb had greater MVIC_dyn-submax_ increase than right upper limb at 90% 1 RM load. The 70% of 1 RM load did not differ bilaterally. This result indicated that at the lighter load allows similar bilateral muscle activity, while very heavy loads require maximal recruitment of motor units in concentric and eccentric action. The results presented in this study have certain limitations. First, the study sample included only one elite athlete. Secondly, the EMG measurements were not repeated within a short time to determine the reliability of results. Thirdly, only two loads (70% 1 RM; 90% 1 RM) and two TUT (2 s/1 s and 6 s/1 s) were evaluated. It would also be interesting to evaluate if post-activation potentiation and pre-exhaustion methods applied to one side of the body modify EMG activity of the prime movers during the BP movement. Additionally, future studies should include measurements of the external structure of the movement (e.g., acceleration, velocity, and displacement).

The novel aspect of our research indicates that while analyzing EMG activity of the prime movers in a symmetrical closed chain movement as the bench press one must considered the activity on both the dominant and non-dominant side of the body. 

## 5. Conclusions

Our results indicate that the use of lighter loads activate muscle groups in a different activation level, potentially allowing for a greater control of these muscle groups. Lifting submaximal and maximal loads causes a simultaneous activation of most motor units involved in the movement. However, the EMG evaluations from elite athletes may not be easily transferrable to the general population, as each experienced athlete may have a stabilized neuromuscular pattern (developed over many years of training) for lifting which has different bilateral activity contribution. 

## Figures and Tables

**Table 1:** Peak muscle activity (%MVIC) of four muscles during the eccentric and concentric phase of the flat bench press with different loads (70% and 90% 1 RM) under 2 times of tension (2 s/1 s and 6 s/1 s).

Muscle	Eccentric Phase				Concentric Phase			
	70% 1 RM—150 kg		90% 1 RM—200 kg		70% 1 RM—150 kg		90% 1 RM—200 kg	
	2 s/1 s	6 s/1 s	2 s/1 s	6 s/1 s	2 s/1 s	6 s/1 s	2 s/1 s	6 s/1 s
Left ADpeak	45 *†‡§	60 †	96 §	89 §	75 †	79 †	157 *‡	171 *
Left PMpeak	24 *†§	27 *†§	57 *§	59 *§	71 *†	77 *†	91 *	90
Left TBlatpeak	75 †	75 †	55 *§	53 *	58 *†‡	81 †	85 *‡	62 *
Left TBlongpeak	36 ‡§	52 *§	36 *§	51 §	92 *	81	90	74
**Left SUM**	180 *	**214**	244 *	252 *	296 *	318 *	424 *	398 *
Right ADpeak	75 ‡	51 †§	87 §	86 §	64 †‡	93 †	135	144
Right PMpeak	48 †‡§	73 †§	91	92	111	106	110	107
Right TBlatpeak	85 §	74	75 §	79 §	137 †‡	86	102	102
Right TBlongpeak	23 †§	32 †§	54 §	63 §	65 †	71	105	95
**Right SUM**	**231**	**232**	**307**	**320**	**377**	**356**	**451**	**448**
**Right/Left difference**	**52**	**18**	**63**	**68**	**81**	**38**	**28**	**50**

Legend: Values have significant differences by range of 20% of MVIC. * differences in right and left side of the body. † differences between 70% and 90% 1 RM. ‡ differences between 2/1 and 6/1 tension. § differences between concentric and eccentric. AD = anterior deltoid, PM = pectoralis major, TB = triceps brachii, lat = lateral head, long = long head, SUM = summary of all primal movers normalized methods.

**Table 2:** Dynamics variability of mean submaximal dynamic contraction (MVIC_dyn-submax_, %) based at 70% 1 RM as a sum of four muscles during the eccentric and concentric phase of the flat bench press with different loads (70% and 90% 1 RM) under 2 times of tension (2 s/1 s and 6 s/1 s).

Muscle	Eccentric Phase				Concentric Phase			
	70% 1 RM—150 kg		90% 1 RM—200 kg		70% 1 RM—150 kg		90% 1 RM—200 kg	
	2 s/1 s	6 s/1 s	2 s/1 s	6 s/1 s	2 s/1 s	6 s/1 s	2 s/1 s	6 s/1 s
**Left**	Basic	+18%	+35% *	+40% *	Basic	+7%	+43% *	+34%
**Right**	Basic	+1%	+75%	+72%	Basic	−5%	+19%	+18%

Legend: * differences in right and left upper limb.

## References

[1] Stastny P., Gołaś A., Blazek D., Maszczyk A., Wilk M., Pietraszewski P., Petr M., Uhlir P., Zając A. (2017). A systematic review of surface electromyography analyses of the bench press movement task. PLoS ONE.

[2] Van den Tillaar R., Saeterbakken A.H., Ettema G. (2012). Is the occurrence of the sticking region the result of diminishing potentiation in bench press?. J. Sports Sci..

[3] Welsch E.A., Bird M., Mayhew J.L. (2005). Electromyographic activity of the pectoralis major and anterior deltoid muscles during three upper-body lifts. J. Strength Cond. Res..

[4] Anderson K.G., Behm D.G. (2004). Maintenance of EMG activity and loss of force output with instability. J. Strength Cond. Res..

[5] Van den Tillaar R., Saeterbakken A. (2014). Effect of fatigue upon performance and electromyographic activity in 6-RM bench Press. J. Hum. Kinet..

[6] Ettema G. (2009). A comparison of successful and unsuccessful attempts in maximal bench pressing. Med. Sci. Sports Exerc..

[7] Van den Tillaar R., Ettema G. (2013). A comparison of muscle activity in concentric and counter movement maximum bench press. J. Hum. Kinet..

[8] Saeterbakken A.H., Mo D.-A., Scott S., Andersen V. (2017). The effects of bench press variations in competitive athletes on muscle activity and performance. J. Hum. Kinet..

[9] Vigotsky A.D., Halperin I., Lehman G.J., Trajano G.S., Vieira T.M. (2017). Interpreting Signal Amplitudes in Surface Electromyography Studies in Sport and Rehabilitation Sciences. Front. Physiol..

[10] Vigotsky A.D., Beardsley C., Contreras B., Steele J., Ogborn D., Phillips S.M. (2017). Greater electromyographic responses do not imply greater motor unit recruitment and ‘hypertrophic potential’ cannot be inferred. J. Strength Cond. Res..

[11] Vigotsky A.D., Ogborn D., Phillips S.M. (2016). Motor unit recruitment cannot be inferred from surface EMG amplitude and basic reporting standards must be adhered to. Eur. J. Appl. Physiol..

[12] Konrad P. (2005). A Practical Introduction to Kinesiological Electromyography.

[13] Enoka R.M., Fuglevand A.J. (2001). Motor unit physiology: Some unresolved issues. Muscle Nerve.

[14] Elliott B.C., Wilson G.J., Kerr G.K. (1989). A biomechanical analysis of the sticking region in the bench press. Med. Sci. Sports Exerc..

[15] Chulvi Medrano I., Diaz Cantalejo A. (2008). Efficacy and safety of the bench press exercise. Review.. Revista Internacional de Medicina y Ciencias de la Actividad Fisica y del Deporte.

[16] Castillo F., Valverde T., Morales A., Pérez-Guerra A., De León F., García-Manso J. (2012). Maximum power, optimal load and optimal power spectrum for power training in upper-body (bench press): A review. Revista Andaluza de Medicina del Deporte.

[17] Maszczyk A., Golas A., Czuba M., Krol H., Wilk M., Kostrzewa M., Zajac A., Ntastny P., Goodwin J. (2016). EMG analysis and modelling of Flat Bench Press using artificial neural networks. S. Afr. J. Res. Sport Phys. Educ. Recreat..

[18] Maszczyk A., Gołaś A., Pietraszewski P., Roczniok R., Zając A., Stanula A. (2014). Application of neural and regression models in sports results prediction. Procedia Soc. Behav. Sci..

[19] Burden A. (2010). How should we normalize electromyograms obtained from healthy participants? What we have learned from over 25 years of research. J. Electromyogr. Kinesiol..

[20] Reiman M.P., Bolgla L.A., Loudon J.K. (2012). A literature review of studies evaluating gluteus maximus and gluteus medius activation during rehabilitation exercises. Physiother. Theory Pract..

[21] Stastny P., Lehnert M., Zaatar A.M., Svoboda Z., Xaverova Z. (2015). Does the Dumbbell-Carrying Position Change the Muscle Activity in Split Squats and Walking Lunges?. J. Strength Cond. Res..

[22] Marras W.S., Davis K., Maronitis A. (2001). A non-MVC EMG normalization technique for the trunk musculature: Part 2. Validation and use to predict spinal loads. J. Electromyogr. Kinesiol..

[23] Gentil P., Oliveira E., Júnior V.D.A.R., Do Carmo J., Bottaro M. (2007). Effects of exercise order on upper-body muscle activation and exercise performance. J. Strength Cond. Res..

[24] Król H., Gołaś A. (2017). Effect of barbell weight on the structure of the flat bench press. J. Strength Cond. Res..

[25] Brennecke A., Guimarães T.M., Leone R., Cadarci M., Mochizuki L., Simão R., Amadio A.C., Serrão J.C. (2009). Neuromuscular activity during bench press exercise performed with and without the preexhaustion method. J. Strength Cond. Res..

[26] Clark R.A., Humphries B., Hohmann E., Bryant A.L. (2011). The influence of variable range of motion training on neuromuscular performance and control of external loads. J. Strength Cond. Res..

[27] Duchateau J., Enoka R.M. (2008). Neural control of shortening and lengthening contractions: Influence of task constraints. J. Physiol..

[28] Flanagan S.D., Mills M.D., Sterczala A.J., Mala J., Comstock B.A., Szivak T.K., DuPont W.H., Looney D.P., McDermott D.M., Hooper D.R. (2014). The relationship between muscle action and repetition maximum on the squat and bench press in men and women. J. Strength Cond. Res..

[29] Sakamoto A., Sinclair P.J. (2012). Muscle activations under varying lifting speeds and intensities during bench press. Eur. J. Appl. Physiol..

[30] Maeo S., Takahashi T., Takai Y., Kanehisa H. (2013). Trainability of muscular activity level during maximal voluntary co-contraction: Comparison between bodybuilders and nonathletes. PLoS ONE.

